# STIM1 Knockout Enhances PDGF-Mediated Ca^2+^ Signaling through Upregulation of the PDGFR–PLCγ–STIM2 Cascade

**DOI:** 10.3390/ijms19061799

**Published:** 2018-06-18

**Authors:** Tzu-Yu Huang, Yi-Hsin Lin, Heng-Ai Chang, Tzu-Ying Yeh, Ya-Han Chang, Yi-Fan Chen, Ying-Chi Chen, Chun-Chun Li, Wen-Tai Chiu

**Affiliations:** 1 Department of Biomedical Engineering, National Cheng Kung University, Tainan 701, Taiwan; sowa010750@gmail.com (T.-Y.H.); wsp90020@gmail.com (Y.-H.L.); blackcatfantasy555@gmail.com (T.-Y.Y.); ovov665@hotmail.com.tw (Y.-H.C.); yingtzai@gmail.com (Y.-C.C.); 2 Institute of Basic Medical Sciences, National Cheng Kung University, Tainan 701, Taiwan; lisa40723@gmail.com (H.-A.C.); fanny870017@hotmail.com (Y.-F.C.); 3 Department of Life Sciences, National Cheng Kung University, Tainan 701, Taiwan; ccli@mail.ncku.edu.tw

**Keywords:** Ca^2+^, STIM1, STIM2, SOCE, PDGF, PLCγ

## Abstract

Platelet-derived growth factor (PDGF) has mitogenic and chemotactic effects on fibroblasts. An increase in intracellular Ca^2+^ is one of the first events that occurs following the stimulation of PDGF receptors (PDGFRs). PDGF activates Ca^2+^ elevation by activating the phospholipase C gamma (PLCγ)-signaling pathway, resulting in ER Ca^2+^ release. Store-operated Ca^2+^ entry (SOCE) is the major form of extracellular Ca^2+^ influx following depletion of ER Ca^2+^ stores and stromal interaction molecule 1 (STIM1) is a key molecule in the regulation of SOCE. In this study, wild-type and STIM1 knockout mouse embryonic fibroblasts (MEF) cells were used to investigate the role of STIM1 in PDGF-induced Ca^2+^ oscillation and its functions in MEF cells. The unexpected findings suggest that STIM1 knockout enhances PDGFR–PLCγ–STIM2 signaling, which in turn increases PDGF-BB-induced Ca^2+^ elevation. Enhanced expressions of PDGFRs and PLCγ in STIM1 knockout cells induce Ca^2+^ release from the ER store through PLCγ–IP3 signaling. Moreover, STIM2 replaces STIM1 to act as the major ER Ca^2+^ sensor in activating SOCE. However, activation of PDGFRs also activate Akt, ERK, and JNK to regulate cellular functions, such as cell migration. These results suggest that alternative switchable pathways can be observed in cells, which act downstream of the growth factors that regulate Ca^2+^ signaling.

## 1. Introduction

Ca^2+^ plays an important and ubiquitous role in intracellular signaling in response to several cellular functions, including cell proliferation, development, differentiation, migration, transcription factor activation, and apoptosis [1,2]. To coordinate these functions, Ca^2+^ signaling is regulated meticulously and is distinguished by several patterns of spatio–temporal parameters with altering amplitude, frequency, or duration [2,3,4]. In cells, Ca^2+^ can be released from the internal store, the endoplasmic reticulum (ER), to increase the intracellular Ca^2+^ concentration. Moreover, there is a common signal transduction pathway to increase cytosolic Ca^2+^ concentration. After the ligand binds to receptor tyrosine kinase (RTK), phospholipase C gamma (PLCγ) is phosphorylated and activated, which in turn hydrolyzes membrane-bound phosphatidylinositol 4,5-bisphosphate (PIP2) into 1,4,5-inositol trisphosphate (IP3) and diacylglycerol (DAG). IP3 can induce the diffusion of Ca^2+^ from the ER into the cytoplasm through the IP3 receptor (IP3R) on the ER membrane. Subsequent exhaustion of ER Ca^2+^ activates a refill mechanism called the store-operated Ca^2+^ entry (SOCE), which also increases cytosolic Ca^2+^ concentration.

SOCE is the major mechanism that increases and replenishes intracellular Ca^2+^ concentration in most non-excitable and a few excitable cells and is triggered following the depletion of ER Ca^2+^ concentration via the store-operated channels on the plasma membrane [5,6,7]. Stromal interaction molecules (STIMs) are distributed on ER membranes as Ca^2+^ store sensors. Following the depletion of ER Ca^2+^ concentration, the activated STIM proteins aggregate near the plasma membrane and interact with two Ca^2+^-permeable components of store-operated channels (SOCs), viz., Ca^2+^ release-activated calcium modulator 1 (CRAM1, also called Orai1) and transient receptor potential canonical 1 (TRPC1), which contribute to SOCE [8,9,10]. Extracellular Ca^2+^ can be passively transported into the cytoplasm through the STIM–Orai1–TRPC1 complex to regulate Ca^2+^-sensitive enzymes and Ca^2+^-dependent transcription factors, such as cAMP response element binding protein (CREB) and nuclear factor of activated T-cells (NFAT) [11,12,13,14].

The STIM family senses ER Ca^2+^ and activates store-operated channels (SOCs) to regulate cell proliferation [15,16] and migration [17,18]. There are two conserved isoforms of STIM proteins, STIM1 and STIM2, which differ from the ER-plasma membrane junction of the STIM structure [19,20,21]. STIM1 has been reported to play a key role in SOCE and regulates SOCE when ER Ca^2+^ store depletes through interacting with Ca^2+^ release-activated calcium modulator 1 (Orai1) in mouse embryonic fibroblasts (MEF) cells [8,22,23]. Previous studies have shown that STIM2 exhibits more sensitivity but lower affinity to Ca^2+^ in the ER lumen than STIM1. Thus, it has been suggested that STIM2 responds to minor elevation in ER luminal Ca^2+^ concentration [19,20,24]. However, the exact role of STIM2 in Ca^2+^ regulation is still unclear. There are three homologues of Orais, viz., Orai1, Orai2, and Orai3, and all three Orai proteins are expressed in plasma membranes and mediate SOCE [25,26,27,28]. TRPC is a non-selective cation channel that works as an SOCE channel. TRPC is classified into seven subtypes, namely TRPC1–TRPC7, all of which respond to PIP2 hydrolysis [9,29]. Of these, TRPC1 is the major subtype involved in STIM-mediated Ca^2+^ influx via the STIM1–Orai1–TRPC1 complex [30,31]. Previous studies have suggested that STIMs, Orais, and TRPC1 play key roles in SOCE, an important mechanism that regulates intracellular Ca^2+^ hemostasis [32,33].

Platelet-derived growth factor (PDGF) is a mitogen that is involved in cellular migration, proliferation, angiogenesis, and extracellular matrix generation [34,35,36,37]. The PDGF family consists of five proteins: PDGF-AA, PDGF-BB, PDGF-AB, PDGF-CC, and PDGF-DD, which are structurally and functionally related and contain a disulfide-linked dimer of two polypeptide chains that are conserved similarly to that in the endothelial growth factor (EGF) family [35,38]. The isoforms bind with different affinities to two receptor units, PDGFRα and PDGFRβ. PDGFRs are composed of two related isoforms of RTKs, namely PDGFRα and PDGFRβ, and form three different subtype receptors, viz., PDGFR-αα, PDGFR-ββ, and PDGFR-αβ, which react with different ligands to initiate various reactions [39,40,41]. Each subtype of PDGF activates different combinations of PDGFRs; however, only PDGF-BB has been verified to bind to all kinds of receptors, resulting in auto-phosphorylation of RTK. The active form of PDGFR activates signaling pathways, such as Akt, JNK, ERK, and STAT3, to regulate cell function and hydrolyzes PIP2 to DAG and IP3 to induce ER Ca^2+^ release [42,43]. Previous studies have shown that PDGF-BB activates PDGFRs (PDGFRα and PDGFRβ) and that the phosphorylation of PDGFR activates PLCγ, which hydrolyze PIP2 to DAG and IP3, leading to a depletion in ER Ca^2+^ store and Ca^2+^ re-entering from the extracellular solution through SOCs [44,45]. Recent studies have also shown that PDGF-BB induces SOCE through STIM1 and Orai1 to promote smooth muscle cell migration [46,47].

Mitogens, such as PDGF and EGF, are well recognized as inducing STIM1-mediated Ca^2+^ elevation including ER Ca^2+^ release and SOCE, for which STIM1 and Orai1, but not STIM2, are essential in PDGF-BB-induced Ca^2+^ elevation and cell migration [16,47,48]. However, STIM2 has reportedly responded to minor decreases in basal Ca^2+^ concentration and replenished Ca^2+^ concentration through the STIM–Orai puncta-like complex [20,24]. In most studies, PDGF-BB has been reported as the SOCE activator that leads to ER Ca^2+^ depletion through the PDGFR–PLCγ–IP3 signaling pathway. However, the role between STIM1 and PDGF-BB signaling is still unclear. Thus, in this study, we employed a particular cell line, mouse embryonic fibroblast-STIM1 knockout cells (MEF-STIM1^−/−^), to study the effects of STIM1 knockout on PDGF-BB-stimulated Ca^2+^ response.

## 2. Results

### 2.1. Stromal Interaction Molecule 1 (STIM1) Knockout Represses Store-Operated Ca^2+^ Entry (SOCE) in Mouse Embryonic Fibroblasts (MEF) Cells

**Figure 1:**
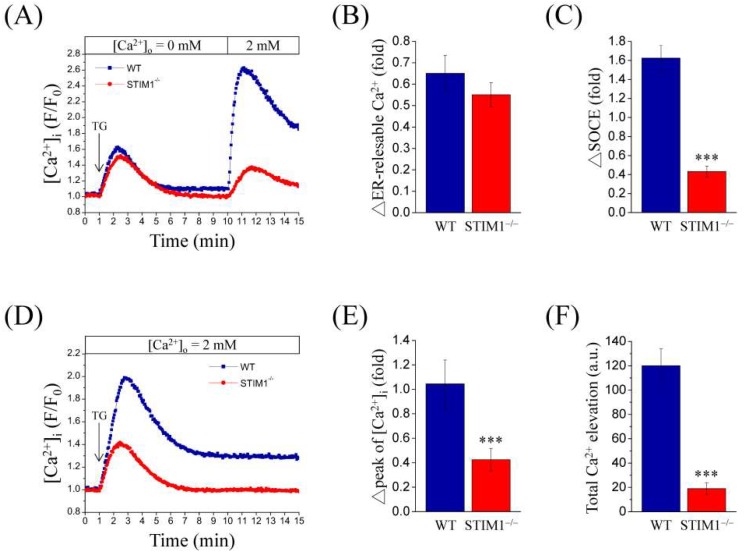
Thapsigargin (TG)-mediated store-operated Ca^2+^ entry (SOCE) is suppressed in mouse embryonic fibroblast-STIM1 knockout (MEF-STIM1^−/−^) cells. (**A**,**D**) Representative tracings show the effect of 2 μM TG (arrow) on Fura-2/AM loaded MEF-WT (wild-type) and MEF-STIM1^−/−^ cells (**A**) in absence of extracellular Ca^2+^ followed by addition of 2 mM Ca^2+^ to the extracellular buffer or (**D**) at 2 mM extracellular Ca^2+^. Intracellular Ca^2+^ ([Ca^2+^]_i_) was monitored using a single-cell fluorimeter for 15 min. Each trace represents the mean of at least four independent experiments. The bar charts show (**B**) ER Ca^2+^ release, (**C**) SOCE, (**E**) initial Ca^2+^ peak (change of peak value), and (**F**) total Ca^2+^ elevation (area under the curve) following the addition of TG. Bars represent mean ± SEM. *** *p* < 0.001 by Student’s *t*-test. TG, thapsigargin; a.u., arbitrary unit.

In order to investigate the effect of STIM1 on SOCE, wild-type (MEF-WT) and STIM1 knockout MEF (MEF-STIM1^−/−^) cell lines were used and intracellular concentration of Ca^2+^ was monitored by using a ratiometric fluorescent dye, Fura-2/AM. Thapsigargin (TG), a sarco/endoplasmic reticulum Ca^2+^-ATPase (SERCA) inhibitor and SOCE activator, is usually used to deplete ER Ca^2+^ store to activate SOCE. The cells were exposed to 2 μM TG in the absence of extracellular Ca^2+^. An initial transient intracellular Ca^2+^ elevation was observed due to Ca^2+^ released from the ER. Next, 2 mM Ca^2+^ was reintroduced into the extracellular solution, leading to Ca^2+^ influx, called SOCE (Figure 1A). Quantification analysis revealed that the ER released Ca^2+^ was similar in both cell types (Figure 1B), but SOCE was significantly reduced in MEF-STIM1^−/−^ cells compared to that in MEF-WT cells (Figure 1C)*.* In addition, cells were exposed to 2 mM extracellular Ca^2+^ and stimulated with 2 μM TG to mimic normal physiological Ca^2+^ concentration. Representative traces indicate a quick two-fold increase in intracellular Ca^2+^ concentration, which then decreased by 1.4-fold in MEF-WT cells. The resultant Ca^2+^ concentration was higher than the baseline and was sustained for a long period. The initial peak indicated that this Ca^2+^ release from the ER was accompanied by Ca^2+^ influx from the extracellular solution, which sustained the higher Ca^2+^ concentration. In MEF-STIM^−/−^ cells, the initial peak was 1.4-fold higher, which then quickly reverted to the baseline concentration (Figure 1D). These results suggest that TG-mediated Ca^2+^ elevation after extracellular 2 mM Ca^2+^ exposure showed an initial peak (Figure 1E) and that the total Ca^2+^ elevation (Figure 1F) in MEF-WT cells was more dominant than that in MEF-STIM1^−/−^ cells. Thus, STIM1 knockout reduced Ca^2+^ elevation in MEF cells, particularly the Ca^2+^ influx.

### 2.2. PDGF-BB Induced Significant Ca^2+^ Elevation in STIM1 Knockout MEF Cells

**Figure 2:**
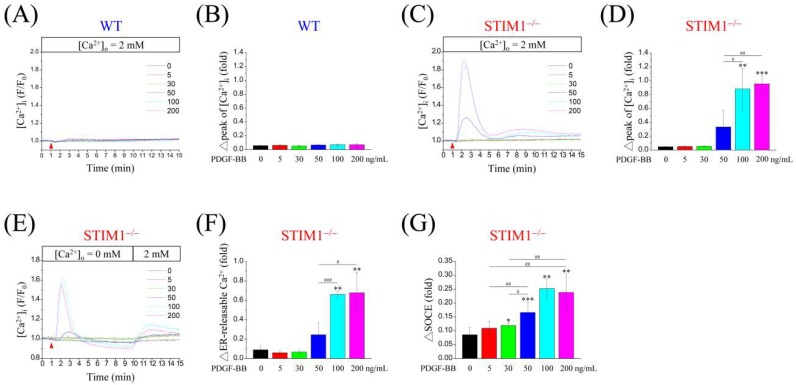
PDGF-BB induces Ca^2+^ elevation in MEF-STIM1^−/−^ cells but not in MEF-WT cells. Representative tracings showing the effect of PDGF-BB (0–200 ng/mL, arrowhead) in Fura-2/AM-loaded, serum-starved (**A**) MEF-WT, (**C**) MEF-STIM1^−/−^ cells at 2 mM extracellular Ca^2+^, and (**E**) MEF-STIM1^−/−^ cells in the absence of extracellular Ca^2+^ followed by addition of 2 mM Ca^2+^ to the extracellular buffer. Intracellular Ca^2+^ ([Ca^2+^]_i_) was monitored using a single-cell fluorimeter for 15 min. (**B**,**D**,**F**,**G**) Bar charts indicate (**B**) initial Ca^2+^ peak of MEF-WT and (**D**) initial Ca^2+^ peak, (**F**) ER Ca^2+^ release, and (**G**) SOCE of MEF-STIM1^−/−^ cells following the addition of PDGF-BB. Bars represent mean ± SEM. *,#: *p* < 0.05; **,##: *p* < 0.01; ***,###: *p* < 0.001 by one-way ANOVA with Dunnett’s post-hoc test.

PDGF promotes cell migration through regulation of Ca^2+^ signaling. Here, we attempted to investigate the effects of STIM1 knockout on Ca^2+^ elevation in MEF cells following PDGF-BB stimulation. Unexpectedly, PDGF-BB induced Ca^2+^ elevation dose-dependently in MEF-STIM1^−/−^ cells (Figure 2C,D) but not in MEF-WT cells (Figure 2A,B). Moreover, quantification analysis of the initial Ca^2+^ revealed that PDGF-BB at 100 ng/mL exerted optimal stimulation in MEF-STIM1^−/−^ cells (Figure 2C) and produced an initial peak that was 1.8-fold higher, which is similar to TG-mediated Ca^2+^ elevation in MEF-WT cells (Figure 1D). In contrast to the TG-mediated Ca^2+^ elevation shown previously, PDGF-BB induced a notable dose-dependent response in MEF-STIM1^−/−^ cells and had no effect in MEF-WT cells. To clarify how PDGF-BB induces Ca^2+^ elevation, intracellularly or extracellularly, MEF-STIM1^−/−^ cells were treated with PDGF-BB (0–200 ng/mL) in the absence of extracellular Ca^2+^, after which 2 mM Ca^2+^ was added to the extracellular solution. There was a transient increase in intracellular Ca^2+^ concentration in MEF-STIM1^−/−^ cells due to the release of ER Ca^2+^, which then reverted to the baseline concentration. Following the addition of 2 mM Ca^2+^, Ca^2+^ influx appeared to replenish the cytosol (Figure 2E). In addition, PDGF-BB at 50 ng/mL triggered substantial Ca^2+^ release from the ER, 100 ng/mL of which was sufficient to induce optimal Ca^2+^ elevation (Figure 2F). More specifically, Ca^2+^ influx analysis with different concentrations of PDGF-BB showed that treatment with 100 ng/mL PDGF-BB induced the highest Ca^2+^ elevation following ER Ca^2+^ depletion (Figure 2G). These data also show that PDGF-BB-mediated Ca^2+^ elevation occurred primarily through intracellular Ca^2+^ release and then through SOCE. Furthermore, knockdown of STIM2 resulted in the downregulation of SOCE upon TG or PDGF-BB treatment in MEF-STIM1^−/−^ cells (Figure S2).

### 2.3. Upregulation and Activation of PDGFRα, PDGFRβ, and Phospholipase C Gamma (PLCγ) in MEF-STIM1^−/−^ Cells

**Figure 3:**
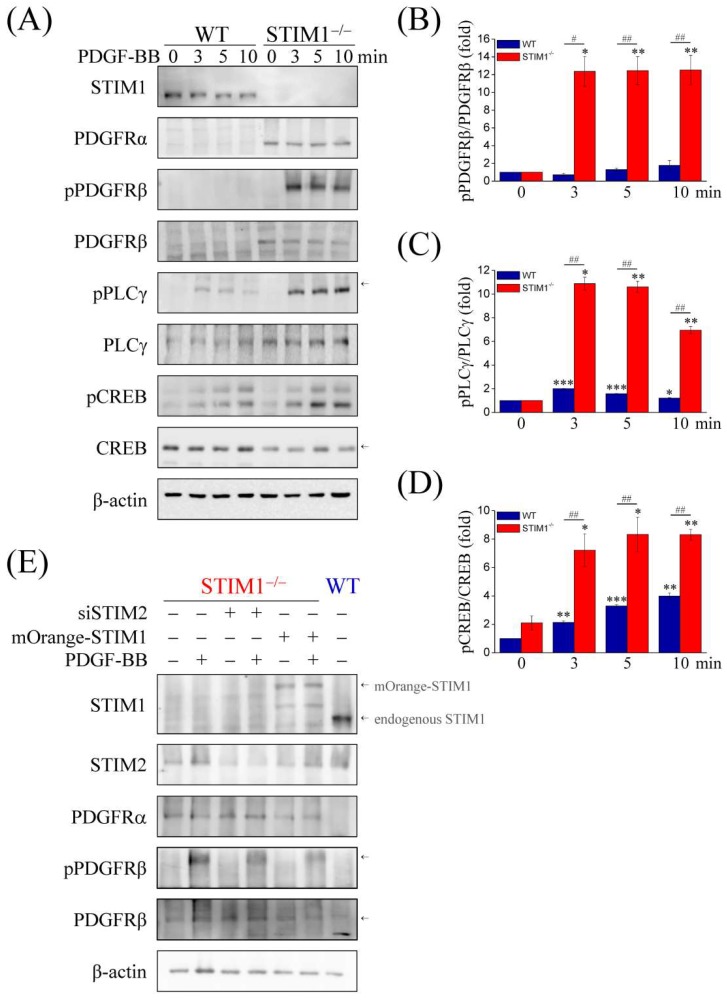
STIM1 knockout enhances the expression of PDGFRα and PDGFRβ, and increases phosphorylation of phospholipase C gamma (PLCγ) and cAMP response element binding protein (CREB). (**A**) MEF-WT and MEF-STIM1^−/−^ cells were starved in a serum-free medium for 12 h and then stimulated with 100 ng/mL PDGF-BB for 3, 5, and 10 min. Immunoblotting analysis using antibodies against STIM1, PDGFRα, phospho-PDGFRβ (pPDGFRβ), PDGFRβ, phospho-PLCγ (pPLCγ), PLCγ, phospho-CREB (CREB), and CREB. β-actin served as the internal control; (**B**–**D**) Measurement of the relative intensities of protein phosphorylation are represented as mean ± SEM from three independent experiments for both MEF-WT and MEF-STIM1^−/−^ cells. Bar charts show phosphorylation levels of (**B**) pPDGFRβ, (**C**) pPLCγ, and (**D**) pCREB, which were normalized to the total protein. *,#: *p* < 0.05; **,##: *p* < 0.01; ***: *p* < 0.001 by Student’s *t*-test; (**E**) Knockdown of STIM2 or overexpression of STIM1 upon transient transfection with STIM2 siRNA (siSTIM2) and mOrange-tagged STIM1 plasmid (mOrange-STIM1) for 48 h in MEF-STIM1^−/−^ cells, respectively. Cells were starved in a serum-free medium for 12 h and then stimulated with or without 100 ng/mL PDGF-BB for 5 min. Immunoblotting analysis using antibodies against STIM1, STIM2, PDGFRα, phospho-PDGFRβ, and PDGFRβ. β-actin served as the internal control. The MEF-WT cells were used as a control for MEF-STIM1^−/−^ cells. Arrows indicate the target proteins.

**Figure 4:**
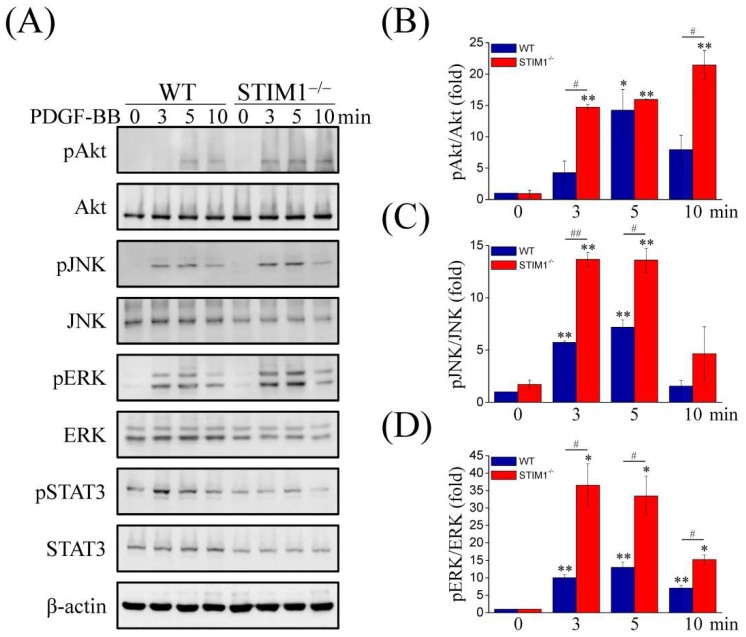
STIM1 knockout increases phosphorylation of Akt, JNK, and ERK but not STAT3 under PDGF-BB stimulation. (**A**) MEF-WT and MEF-STIM1^−/−^ cells were starved in a serum-free medium for 12 h and then stimulated with 100 ng/mL PDGF-BB for 3, 5, and 10 min. Immunoblotting analysis using antibodies against phospho-Akt (pAkt), Akt, phospho-JNK (pJNK), JNK, phospho-ERK (pERK), ERK, phospho-STAT3 (pSTAT3), and STAT3. β-actin served as the internal control; (**B**–**D**) Measurement of the relative intensities of protein phosphorylation are represented as mean ± SEM from three independent experiments for both MEF-WT and MEF-STIM1^−/−^ cells. Bar charts show the phosphorylation levels of (**B**) pAkt, (**C**) pJNK, and (**D**) pERK, which were normalized to the total protein. *,#: *p* < 0.05; **,##: *p* < 0.01 by Student’s *t*-test.

Previous studies have shown that PDGF-BB activates PDGFRs (PDGFRα and PDGFRβ) and that PDGFR phosphorylation activates PLCγ to hydrolyze PIP2 into DAG and IP3, which leads to a depletion of the ER Ca^2+^ store. Therefore, we examined PDGF-BB-mediated signaling pathways. Immunoblotting showed that expressions of PDGFRα, PDGFRβ, and PLCγ were enhanced in MEF-STIM1^−/−^ cells compared to those in MEF-WT cells (Figure 3A), indicating that the upregulation was due to PDGF-BB stimulation. Quantification analyses of the ratio of phosphorylated PDGFRβ:PDGFRβ (Figure 3B) and phosphorylated PLCγ:PLCγ (Figure 3C) also confirmed the results, because their activities following PDGF-BB treatment were evidently increased in MEF-STIM1^−/−^ cells compared to those in MEF-WT cells. CREB activation by phosphorylation can be triggered by both PDGF and Ca^2+^ signal transduction pathways and inhibition of CREB expression or activation decreases PDGF-induced smooth muscle cell migration. Thus, we examined the phosphorylation of CREB in response to PDGF-BB stimulation. The results showed that CREB was phosphorylated in MEF-STIM1^−/−^ cells and the phosphorylation levels were higher than those in MEF-WT cells (Figure 3D). STIM2 knockdown did not affect the expressions of PDGFRα and PDGFRβ and the PDGF-BB-induced PDGFRβ phosphorylation, whereas STIM1 overexpression downregulated the expressions of PDGFRα and PDGFRβ and the PDGF-BB-induced PDGFRβ phosphorylation (Figure 3E). We then sought to determine other non-Ca^2+^-conducting PDGF-BB-induced downstream signaling molecules, including Akt, JNK, ERK and STAT3 (Figure 4A). Upon PDGF-BB stimulation, Akt phosphorylation increased within 3 min in MEF-STIM1^−/−^ cells and was sustained for at least 10 min; however, in MEF-WT cells, Akt was activated within 5 min and then decreased quickly (Figure 4B). Although phosphorylation of JNK was triggered by PDGF-BB in both cell types, the levels of phosphorylation were higher in MEF-STIM1^−/−^ cells than those in the MEF-WT cells (Figure 4C). In addition, PDGF-BB induced higher levels of ERK phosphorylation in MEF-STIM1^−/−^ cells than that in MEF-WT cells (Figure 4D). Activation of STAT3 upon PDGF-BB stimulation was not significantly different between MEF-WT and MEF-STIM1^−/−^ cells. Taken together, these findings support the responses of PDGF-BB-induced Ca^2+^ elevation in MEF-STIM1^−/−^ cells due to the elevated protein levels of PDGFRs, resulting in higher activation of PLCγ, Akt, JNK, and ERK than in MEF-WT cells. PLCγ was the key regulator upstream of IP3-mediated ER Ca^2+^ release and SOCE. The PDGFR kinase inhibitor AG1295 was used to verify the role of PDGFR activation in PDGF-BB-induced PLCγ signaling (Figure S1A). Our results showed that inhibition of PDGFRβ activation using AG1295 (Figure S1B) resulted in cessation of PDGF-BB-mediated PLCγ signaling in MEF-STIM1^−/−^ cells (Figure S1C). Moreover, PLC inhibitors U73122 and D609 were used to examine PDGF-BB-induced Ca^2+^ responses, such as CREB phosphorylation (Figure S3A). These results indicated that PLC inhibitors did not affect PDGFR phosphorylation upon PDGF-BB stimulation (Figure S3A) but inhibited phosphorylation of PLCγ and CREB (Figure S3B,C).

### 2.4. PDGF-BB-Mediated ER Store-Depletion Activates STIM2 Translocation and Puncta Formation

**Figure 5:**
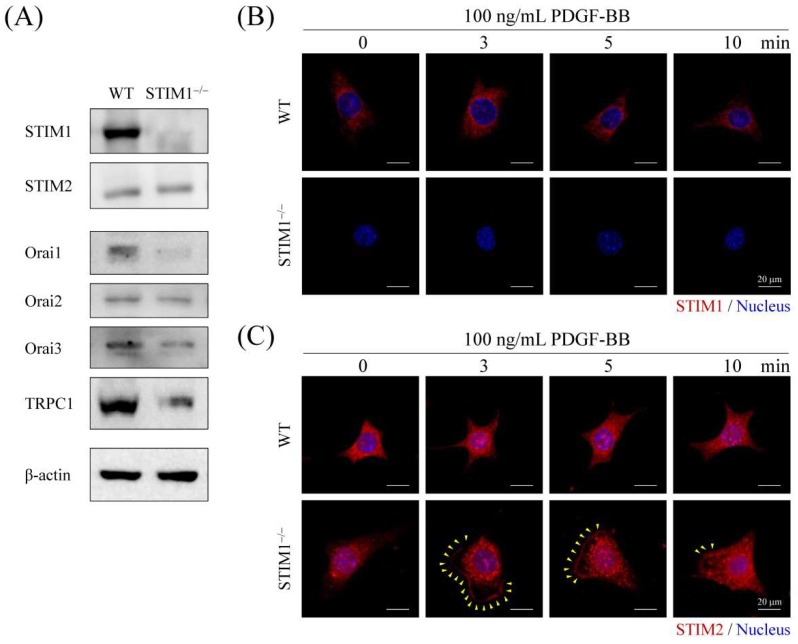
PDGF-BB induces STIM2 puncta formation and translocation in MEF-STIM1^−/−^ cells. (**A**) STIM1, STIM2, Orai1, Orai2, Orai3 and transient receptor potential canonical 1 (TRPC1) were detected using immunoblotting in both MEF-WT and MEF-STIM1^−/−^ cells. β-actin served as the internal control; (**B**,**C**) MEF-WT and MEF-STIM1^−/−^ cells were starved in a serum-free medium and then stimulated with 100 ng/mL PDGF-BB for 3, 5, and 10 min. Then, cells were processed for immunofluorescence staining using (**B**) anti-STIM1 or (**C**) anti-STIM2 antibody, followed by adding a secondary antibody coupled to Alexa Fluor^®^ 594 dye and Hoechst 33342 (blue) was used as the nuclear marker. Fluorescence images were captured using a laser scanning confocal microscope. Yellow arrowheads indicate plasma membrane translocation of the STIM2 protein. Scale bars = 20 μm.

**Figure 6:**
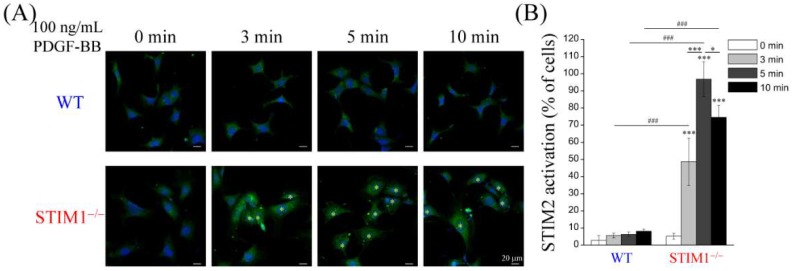

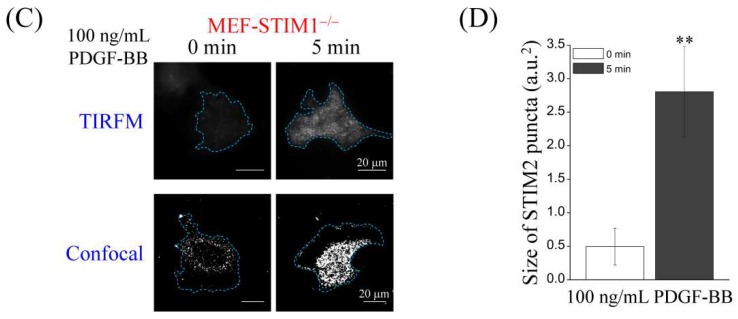
PDGF-BB induces STIM2 activation in MEF-STIM1^−/−^ cells. (**A**) MEF-WT (WT) and MEF-STIM1^−/−^ (STIM1^−/−^) cells were starved in a serum-free medium and then stimulated with 100 ng/mL PDGF-BB for 3, 5, and 10 min. Then, cells were processed for immunofluorescence staining using anti-STIM2 antibody, followed by adding a secondary antibody coupled to Alexa Fluor^®^ 488 dye. Fluorescence images were captured using a laser scanning confocal microscope. Yellow stars indicate cells with activated STIM2, presented as puncta formation and plasma membrane translocation. Scale bars = 20 μm; (**B**) Quantitative analysis of PDGF-BB-induced STIM2 activation that was assessed from the STIM2 plasma membrane translocated cells from three independent experiments. Bars represent mean ± SEM. *: *p* < 0.05; ***,###: *p* < 0.001 by Student’s *t*-test; (**C**) MEF-STIM1^−/−^ cells were starved in a serum-free medium and then stimulated with 100 ng/mL PDGF-BB for 5 min. Then, cells were processed for immunofluorescence staining using an anti-STIM2 antibody, followed by adding a secondary antibody coupled to Alexa Fluor^®^ 488 dye. Fluorescence images were captured using a total internal reflection fluorescence microscope or laser scanning confocal microscope. Blue dashed lines indicate the periphery of cells. Scale bars = 20 μm; (**D**) Quantitative analysis of PDGF-BB-induced aggregation of STIM2 puncta from three independent experiments (*n* > 30 cells). Bars represent mean ± SEM. **: *p* < 0.01 by Student’s *t*-test.

STIMs, Orais, and TRPC1 play key roles in the regulation of SOCE. As shown in Figure 2, PDGF-BB induced SOCE in MEF-STIM1^−/−^ cells but not in MEF-WT cells. To understand how PDGF-BB induced Ca^2+^ elevation in MEF-STIM1^−/−^ cells, proteins that contribute to SOCE were examined by immunoblotting analysis. Although STIM1 knockout did not affect STIM2; Orai1, Orai2, Orai3, and TRPC1 were decreased in MEF-STIM1^−/−^ cells (Figure 5A). These results supported the observation that Ca^2+^ influx following TG-mediated ER store depletion in MEF-STIM1^−/−^ cells is lower than that in MEF-WT cells because of lower SOCE-associated protein levels in MEF-STIM1^−/−^ cells (Figure 1). To investigate the role of STIM proteins in MEF cells in response to PDGF-BB stimulation, the subcellular localization of endogenous STIM1 and STIM2 was examined by immunostaining. The confocal images showed that STIM1 was not expressed in MEF-STIM1^−/−^ cells (Figure 5B). Moreover, the STIM2 protein translocated near the plasma membrane and formed distinct intracellular puncta in MEF-STIM1^−/−^ cells after PDGF-BB treatment from 3 to 10 min. Maximal activity was observed at 5 min, after which it decreased over time. In contrast, PDGF-BB did not induce puncta formation and plasma membrane translocation of STIMs in MEF-WT cells (Figure 5C and Figure 6A,B ). In addition, total internal reflection fluorescence microscopy (TIRFM) and high magnification laser scanning confocal microscopy were applied to verify plasma membrane translocation and intracellular puncta formation of activated STIM2, respectively. TIRFM is a technique used to record the fluorescence signals within 50 to 100 nm range of the basal plasma membrane. We observed a significant plasma membrane translocation of STIM2 after PDGF-BB stimulation for 5 min in MEF-STIM1^−/−^ cells (Figure 6C). On the other hand, large size of the internal aggregation of the STIM2 puncta was presented in PDGF-BB stimulated MEF-STIM1^−/−^ cells (Figure 6C). Quantitative results showed that PDGF-BB induced 5–6-fold increases of STIM2 aggregation (Figure 6D). We also demonstrated colocalization between SITM2 and Orai1, Orai2, Orai3, or TRPC1 after PDGF-BB treatment (Figure S4).

### 2.5. SOCE Inhibitors Decrease PDGF-BB-Induced Ca^2+^ Elevation in MEF-STIM1^−/−^ Cells

**Figure 7:**
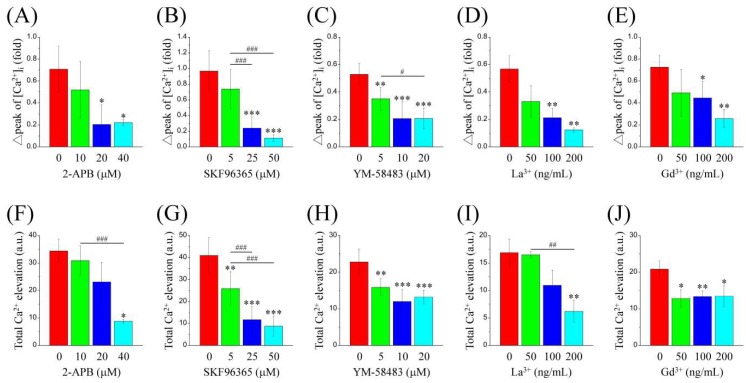
Dose-response inhibition of PDGF-BB-mediated Ca^2+^ elevation by the Ca^2+^ mobilization inhibitors. MEF-STIM1^−/−^ cells were starved in a serum-free medium and then loaded with Fura-2/AM and co-treated with (**A**,**F**) 0–40 μM 2-APB, (**B**,**G**) 0–50 μM SKF96365, (**C**,**H**) 0–20 μM YM58483, (**D**,**I**) 0–200 ng/mL La^3+^, and (**E**,**J**) 0–200 ng/mL Gd^3+^ for 30 min. Cells were then stimulated with 100 ng/mL PDGF-BB at 2 mM extracellular Ca^2+^. Intracellular Ca^2+^ ([Ca^2+^]_i_) was monitored using a single-cell fluorimeter for 15 min for at least three independent experiments. The bar charts show (**A**–**E**) initial Ca^2+^ peak and (**F**,**J**) total Ca^2+^ elevation following the addition of PDGF-BB. Bars represent mean ± SEM. *,#: *p* < 0.05; **,##: *p* < 0.01; ***,###: *p* < 0.001 by one-way ANOVA with Dunnett’s post-hoc test.

Pharmacological inhibitors of SOCE, such as 2-APB (Figure 7A), SKF96365 (Figure 7B), YM-58483 (Figure 7C), La^3+^ (Figure 7D), and Gd^3+^ (Figure 7E) were used to examine the role of Ca^2+^ mobilization in PDGF-induced Ca^2+^ elevation in MEF-STIM1^−/−^ cells exposed to 2 mM Ca^2+^ solution. Quantification analysis of the initial Ca^2+^ and total Ca^2+^ elevation following PDGF-BB stimulation showed that Ca^2+^ signaling was inhibited by these inhibitors in a dose-response manner (Figure 7).

### 2.6. PDGFR–PLCγ–STIM2 Signaling Induces Cell Migration in MEF-STIM1^−/−^ Cells.

**Figure 8:**
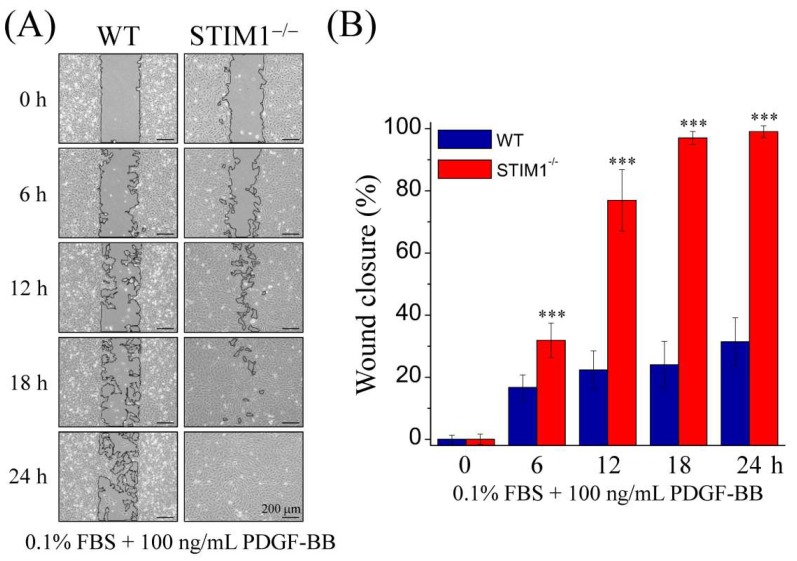
PDGF-BB induces cell migration in MEF-STIM1^−/−^ cells. (**A**) MEF-WT and MEF-STIM1^−/−^ cells were seeded into silicon inserts with 0.5% fecal bovine serum (FBS) medium. Following cell adhesion, inserts were removed, followed by the addition of Dulbecco’s modified Eagle’s medium (DMEM) with 0.1% FBS + PDGF-BB (100 ng/mL) for 24 h. Phase images were captured using inverted phase-contrast microscopy and wound spaces were analyzed using ImageJ; (**B**) Comparison of the effects of PDGF-BB-induced wound closure following insert removal in MEF-WT and MEF-STIM1^−/−^ cells. Cellular migratory ability is presented as the percentages of wound closure. Bars represent mean ± SEM. ***: *p* < 0.001 by Student’s *t*-test. Scale bars = 200 μm.

PDGF signaling regulates cell proliferation and migration. To determine the influence of PDGF-BB stimulation in MEF-STIM1^−/−^ cells, we performed a wound healing assay and cell count with Hoechst 33342 nuclear staining to examine cell migration and proliferation, respectively. To minimize the effect of serum, we reduced the concentration of serum to 0.1%. By comparing MEF-WT and MEF-STIM1^−/−^ cells, it was observed that the proliferation rate of MEF-WT cells was higher than that of MEF-STIM1^−/−^ cells and that the number of MEF-STIM1^−/−^ cells decreased slowly after 24 h (Figure S5A). To avoid cell proliferation interference, cells were treated with 0.1% fecal bovine serum (FBS) as a control and observed over 24 h. We found that 100 ng/mL PDGF-BB with 0.1% FBS did not affect the proliferation of MEF cells (Figure S5B). In addition, MEF-STIM1^−/−^ cells treated with PDGF-BB led to a substantial increase in wound closure, wherein the wound healed completely after 18 h, unlike MEF-WT cells (Figure 8). These data indicated that STIM1 knockout enhances cell migration when compared with MEF-WT cells.

## 3. Discussion

STIM1 plays the major role in controlling Ca^2+^ homeostasis through regulation of SOCE in many non-excitable cells. In this study, we found the unexpected result that PDGF-BB induced higher intracellular Ca^2+^ elevation in MEF-STIM1^−/−^ cells than that in the parental MEF-WT cells (Figure 2). The observed results were associated with STIM2-dependent SOCE (Figure S2). Immunoblotting revealed that PDGFRα, PDGFRβ, and PLCγ were overexpressed in MEF-STIM1^−/−^ cells and higher PDGFR and PLCγ phosphorylation was observed after PDGF-BB stimulation (Figure 3). In contrast, the levels of both PDGFRα and PDGFRβ were very low in MEF-WT cells, and hence, PDGF-BB could not induce PDGFR phosphorylation and Ca^2+^ elevation (Figure 2 and Figure 3). Increased PLCγ activation may increase the propensity of IP3 to activate the IP3 receptors and lead to Ca^2+^ leakage from the ER lumen, which is the major source of PDGF-BB-mediated Ca^2+^ elevation in MEF-STIM1^−/−^ cells (Figure 2). Depletion of the ER Ca^2+^ store through PDGFR–PLCγ axis following extracellular Ca^2+^ influx is termed as SOCE. However, STIM1 was not expressed in MEF-STIM1^−/−^ cells and Ca^2+^ influx following the re-introduction of 2 mM Ca^2+^ was lower than that of TG-induced SOCE in MEF-WT cells (Figure 1 and Figure 2).

PDGFR (AG1295) and PLC (U73122 and D609) inhibitors were employed to verify whether the PDGFR–PLCγ axis could be activated by PDGF-BB stimulation in MEF-STIM1^−/−^ cells [49,50,51]. Immunoblotting results supported the essential roles of PDGFRs and PLCγ in PDGF-BB-mediated activation (Figures S1 and S3). In addition, the Ca^2+^-activated transcription factor CREB was also observed in correspondence to activation of PDGFR–PLCγ axis-mediated signaling. These results indicated that PDGF-BB increased Ca^2+^ elevation through the activation of PLCγ.

SOCE inhibitors manifestly lowered intracellular Ca^2+^ elevation in a dose-response manner (Figure 7). Accordingly, SOCE may contribute to PDGF-BB-mediated Ca^2+^ elevation upon PDGF-BB stimulation. Here, we confirmed that STIM2 activation was observed in MEF-STIM1^−/−^ cells but not in MEF-WT cells (Figure 5 and Figure 6). PDGF-BB induced interaction between STIM2 and SOCE-related channel proteins in MEF-STIM1^−/−^ cells (Figure S4). However, the level of PDGF-BB-mediated SOCE were lower than ER released Ca^2+^ in MEF-STIM1^−/−^ cells (Figure 1 and Figure 2), which may be due to the downregulation of SOCs Orais and TRPC1 (Figure 2C and Figure 5A). Our results demonstrated that enhancement of Ca^2+^ signaling in MEF-STIM1^−/−^ cells was mediated by the PDGFR–PLCγ–STIM2 pathway.

Proliferation assay of MEF cells also indicated that MEF-STIM1^−/−^ cells proliferated more slowly than MEF-WT cells [22]. Moreover, we showed that MEF-WT cells proliferated more quickly than MEF-STIM1^−/−^ cells, even in a low-serum concentration containing 0.1% FBS, whereas PDGF-BB did not affect MEF proliferation in the same condition (Figure S5). Furthermore, our results showed that the ability of cell migration with PDGF-BB treatment in MEF-STIM1^−/−^ cells was higher than that in MEF-WT cells, regardless of the low cell proliferation rate. It may be that STIM1 knockout evoked migration via PDGFR–PLCγ–STIM2 enhancement, as demonstrated by the wound healing assay (Figure 8). PDGF-BB-induced Ca^2+^ elevation may promote MEF-STIM1^−/−^ cell migration as well as activation of Akt, ERK, and JNK, which could also participate in cell migration regulation. Both EGF and PDGF share similar signal pathways, regulate many similar functions of the cell, and induce SOCE following ER Ca^2+^ depletion through PLCγ signaling [47,52]. We also observed EGFR downregulation in MEF-STIM1^−/−^ cells. Moreover, EGF induced higher activity of EGFR, PLCγ, Akt, JNK, and ERK in MEF-WT cells compared to those in MEF-STIM1^−/−^ cells (Figure S6). PDGF-BB induced higher response in MEF-STIM1^−/−^ cells, whereas EGF induced higher response in MEF-WT cells. These contradictions resulted from a switch in these different expressions of EGFR and PDGFR.

STIM1 has been vigorously studied and is well-established in response to high Ca^2+^ leakage from the ER lumen, which leads to huge and constitutive Ca^2+^ influx through interacting with Orai1 and TRPC1 in the plasma membrane [8,53]. Without STIM1, STIM2 replaces the role of STIM1 and induces lower Ca^2+^ influx [54]. A recent study shows the essential role of STIM proteins in Ca^2+^ homeostasis and their crucial role in controlling multiple functions of smooth muscle cells [12]. The critical role of STIM1 can be partially rescued by STIM2. STIM1 and Orai1, but not STIM2, are essential for PDGF-BB-induced Ca^2+^ elevation and cell migration in human airway smooth muscle cells, which express both STIM proteins [47]. Our results also showed that STIM1 played a curial role in response to store depletion after TG treatment (Figure 1). Interestingly, we found that PDGF-BB induced Ca^2+^ elevation in MEF-STIM1^−/−^ cells but not in MEF-WT cells. STIM2 seemed to partially replace the regulatory role of SOCE by STIM1. To our knowledge, this is the first report that shows enhanced Ca^2+^ elevation in MEF-STIM1^−/−^ cells compared to that in MEF-WT cells because of overexpression of PDGFRs and PLCγ in MEF-STIM1^−/−^ cells.

## 4. Materials and Methods

### 4.1. Cell Culture and Reagents

Wild-type (MEF-WT) and STIM1 knockout (MEF-STIM1^−/−^) mouse embryonic fibroblasts were maintained in high-glucose Dulbecco’s modified Eagle’s medium (DMEM; Caisson, Smithfield, VA, USA) supplemented with 5% fetal bovine serum (FBS; GIBCO, Big Cabin, OK, USA), 100 IU/mL penicillin, 100 μg/mL streptomycin, non-essential amino acid (NEAA; GIBCO), and 1 mM sodium pyruvate (NEAA; GIBCO) under 5% CO_2_ at 37 °C. Recombinant mouse PDGF-BB and EGF were purchased from R&D Systems (Minneapolis, MN, USA). 

### 4.2. Single-Cell Ca^2+^ Measurement

Intracellular Ca^2+^ was measured by the ratiometric Fura-2 fluorescence method using a single-cell fluorimeter (Till Photonics, Grafelfing, Germany). Fura-2 were excited alternatively between 340 nm and 380 nm using the Polychrome IV monochromator (Till Photonics), which is highly sensitive and can detect rapid changes in Ca^2+^ concentration with high temporal and spatial resolution and images were captured using an Olympus IX71 inverted microscope equipped with a xenon illumination system and an IMAGO CCD camera (Till Photonics). Emission fluorescence intensity of 510 nm was used to calculate intracellular Ca^2+^ levels using the TILLvisION 4.0 software (Till Photonics).

### 4.3. Immunoblotting Analyses

Cell lysates were harvested in radioimmunoprecipitation assay (RIPA) buffer (150 mM NaCl, 1 mM EGTA, 50 mM Tris at pH 7.4, 10% glycerol, 1% Triton X-100, 1% sodium deoxycholate, 0.1% SDS, and CompleteTM), and the lysates were analyzed by Western blotting using antibodies against STIM1 (BD, Franklin Lakes, NJ, USA), Orai1, Orai3 (ProSci, Poway, CA, USA), Orai2 (Enzo, Farmingdale, NY, USA), TRPC1 (Proteintech, Rosemont, IL, USA), PDGFRα, JNK, phospho-EGFR, EGFR (Santa Cruz, Santa Cruz, CA, USA), phospho-PDGFRβ, PDGRβ (Abnova, San Francisco, CA, USA), STIM2, phospho-PLCγ, PLCγ, phospho-Akt, Akt, phospho-JNK, phospho-ERK, ERK, phospho-STAT3, STAT3, phospho-CREB, CREB (Cell Signaling, Beverly, MA, USA), and β-actin (Sigma, Saint Louis, MO, USA). The immune complexes were then detected with horseradish peroxidase-conjugated IgG (Jackson ImmunoResearch Laboratories, West Grove, PA, USA), and the reaction was developed using an enhanced chemiluminescence (ECL) detection kit under an ImageQuant LAS 4000 system (GE Healthcare Life Sciences, Pittsburgh, PA, USA).

### 4.4. Immunofluorescence Staining and Confocal Microscopy

MEF cells (3 × 10^4^ cells) were seeded in a 3-cm glass-bottom dish (Alpha Plus, Taoyuan, Taiwan). After adhesion, cells were starved in a serum-free medium for 12 h and then treated with PDGF-BB (100 ng/mL) for 3, 5, and 10 min. Then, cells were fixed with 4% paraformaldehyde (Alfa Aesar, Haverhill, MA, USA) for 10 min; incubated in PBS washing buffer containing 0.1% Triton X-100 for 10 min; and then in CAS-Block solution (Invitrogen, Carlsbad, CA, USA) for 1 h with gentle shaking. Subsequently, the cells were loaded with primary antibody, anti-STIM1 (Cell Signaling) and anti-STIM2 (Alomone Labs, Jerusalem, Israel) diluted to 1:100 in filtered PBS. Finally, cells were stored overnight at 4°C. Alexa Fluor-conjugated secondary antibodies (Invitrogen) were used to counteract primary antibodies, and Hoechst 33342 was loaded as the nuclear marker. The fluorophores were excited by laser at 405 and 594 nm and detected by a laser scanning confocal microscope (FV-1000, Olympus, Tokyo, Japan). The FV10-ASW software (version 4.2a, Tokyo, Japan) was used to access puncta formation and translocation of the STIM proteins.

### 4.5. Wound Healing Assay

Culture inserts (ibidi) were applied to assess MEF cell migration. The insert consisted of two wells separated by a 500 μm-thick silicon wall. The culture insert was placed into a 3-cm culture dish and slightly pressed on the top of the insert to ensure adhesion on the dish. MEF-WT and MEF-STIM1^−/−^ cells were seeded at an equal density (3 × 10^4^ cells in 100 µL) with 0.5% FBS medium and incubated at 37 °C with 5% CO_2_ overnight. The insert was removed after cells were well-attached and formed a monolayer. The cells were then incubated in DMEM containing 0.1% FBS with 100 ng/mL PDGF-BB. Cell migrating into the gap (initially ~500 μm) was recorded every 6 h via phase-contrast microscopy. The data were collected from three independent experiments and analyzed as wound closure (%) by the ImageJ software (version 1.47, Bethesda, MD, USA).

### 4.6. Cell Proliferation Analysis

To assess MEF cell proliferation, cells were stained using Hoechst 33342. MEF cells (WT and STIM1^−/−^) were seeded into a 3-cm culture dish with 0.5% FBS medium for cell starvation and incubated at 37 °C and 5% CO_2_ overnight. After cells had attached properly to the dish, the medium was changed (0.1% FBS with 100 ng/mL PDGF-BB). The cells were collected and fixed with 4% paraformaldehyde at 0, 24, 48, and 72 h. Next, the cells were stained with Hoechst 33342 for 30 min. Fluorescence images were captured randomly and counted using fluorescence microscopy and ImageJ software, respectively.

### 4.7. Statistical Analysis

All data were reported as mean ± SEM (standard error of the mean). For statistical analysis, Student’s *t*-test or one-way ANOVA with Dunnett’s post-hoc test were processed using the Origin software, for which differences were considered significant when *p*-value was <0.05.

## Supplementary Materials

Supplementary materials can be found at http://www.mdpi.com/1422-0067/19/6/1799/s1.

## Abbreviations

MEF	Mouse embryonic fibroblasts
PDGF	Platelet-derived growth factor
PDGFR	Platelet-derived growth factor receptor
SOCE	Store-operated Ca^2+^ entry
STIM1	Stromal interaction molecule 1
Orai1	Ca^2+^ release-activated calcium modulator 1
TRPC1	Transient receptor potential canonical 1
PLCγ	Phospholipase C gamma
RTK	Receptor tyrosine kinase
CREB	cAMP response element binding protein
