# Supplementary material for: STIM1 Knockout Enhances PDGF-Mediated Ca2+ Signaling through Upregulation of the PDGFR–PLCγ–STIM2 Cascade
Source: Int J Mol Sci. 2018 Jun 18;19(6):1799. doi: 10.3390/ijms19061799 (PMC6032054; doi:10.3390/ijms19061799)
Supplement: Supplementary file 1 [file ijms-19-01799-s001.pdf]

Supplementary information

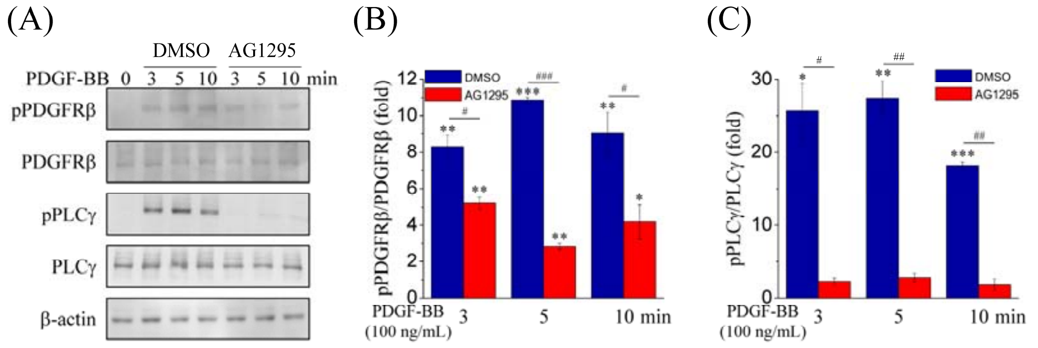

Figure S1. PDGFR inhibitor represses PDGF-BB-induced phosphorylation of PDGFR and PLCγ. MEF-STIM1$^{-/-}$ cells were starved in a serum-free medium for 12 h and then pretreated with 10 μM AG-1295 for 30 min. Cells were stimulated with 100 ng/mL PDGF-BB for 3, 5, and 10 min. Immunoblotting analysis using antibodies against phospho-PDGFRβ (pPDGFRβ), PDGFRβ, phospho-PLCγ (pPLCγ), and PLCγ. β-actin served as the internal control. (B and C) Quantification of proteins phosphorylation between DMSO and AG-1295 treatments. Relative intensities of the blots are presented as mean ± SEM from three independent experiments. Bar charts show phosphorylation levels of (B) pPDGFRβ and (C) pPLCγ normalized to the total protein. *,#: $P < 0.05$; **,##: $P < 0.01$; ***,###: $P < 0.001$ by Student's *t*-test.

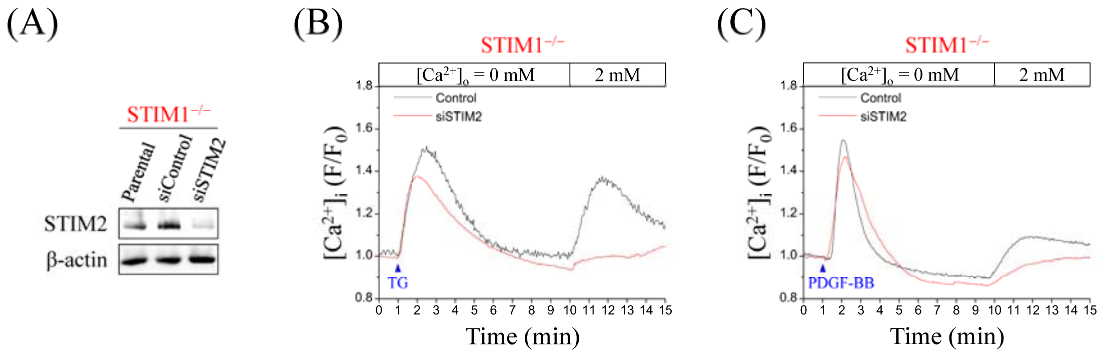

Figure S2. STIM2 knockdown attenuates SOCE in MEF-STIM1$^{-/-}$ cells. (A) Western blotting of STIM2 and β-actin was performed to evaluate the knockdown efficiency of scrambled siRNA (siControl) and STIM2 siRNA (siSTIM2) upon pre-treatment with siRNAs for 48 h in MEF-STIM1$^{-/-}$ cells. Representative tracings showing the effect of STIM2 knockdown in Fura-2/AM-loaded, serum-starved MEF-STIM1$^{-/-}$ cells in the absence of extracellular Ca^{2+} followed by addition of 2 mM Ca^{2+} to the extracellular buffer under stimulated with (B) thapsigargin (TG, 2 μM) or (C) PDGF-BB (100 ng/mL). Intracellular Ca^{2+} ($[Ca^{2+}]_i$) was monitored using a single-cell fluorimeter for 15 min. Each trace represents the mean from three independent experiments. Blue arrowheads indicate addition of TG or PDGF-BB at 1 min.

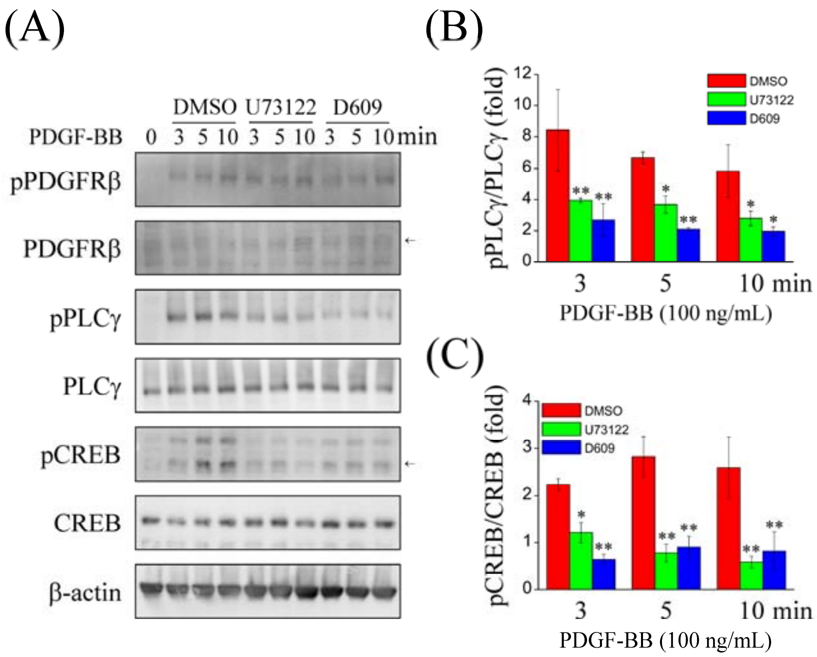

Figure S3. Inhibition of PDGF-BB-mediated PLCγ activation using the PLC inhibitor. MEF-STIM1$^{-/-}$ cells were starved in a serum-free medium for 12 h and then pretreated with 10 μM U73122 and 100 ng/mL D609 for 30 min. Cells were stimulated with 100 ng/mL PDGF-BB for 3, 5, and 10 min. Immunoblotting analysis using antibodies against phospho-PDGFRβ (pPDGFRβ), PDGFRβ, phospho-PLCγ (pPLCγ), PLCγ, phospho-CREB (pCREB), and CREB. β-actin served as the internal control. (B and C) Quantification of protein phosphorylation between DMSO, U73122, and D609 treatments. Relative intensities of the blots are presented as mean ± SEM from three independent experiments. Bar charts show phosphorylation levels of (B) pPLCγ and (C) pCREB normalized to the total protein. *: $P < 0.05$; **: $P < 0.01$ by Student's *t*-test.

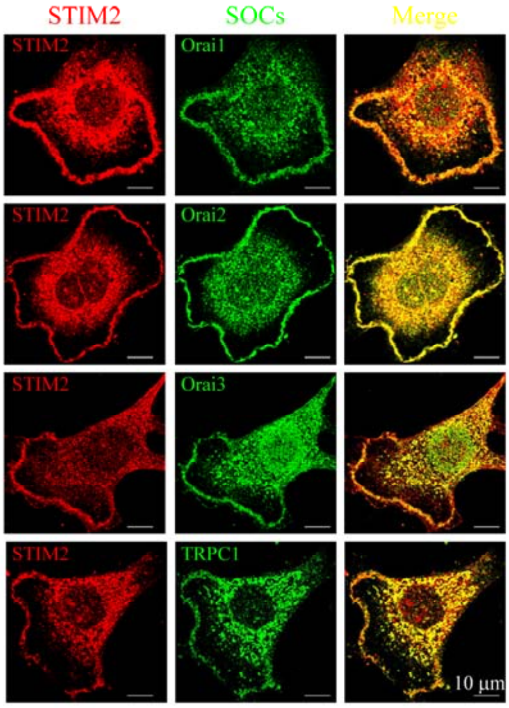

Figure S4. PDGF-BB induces interaction between STIM2 and SOCE-related channel proteins in MEF-STIM1$^{-/-}$ cells. Starved cells were treated with 100 ng/mL PDGF-BB for 5 min, and cells were fixed with 4% paraformaldehyde. Immunofluorescence staining was performed to label STIM2, Orai1, Orai2, Orai3, and TRPC1, and the fluorescence images were obtained using confocal microscopy. Scale bars = 10 μm.

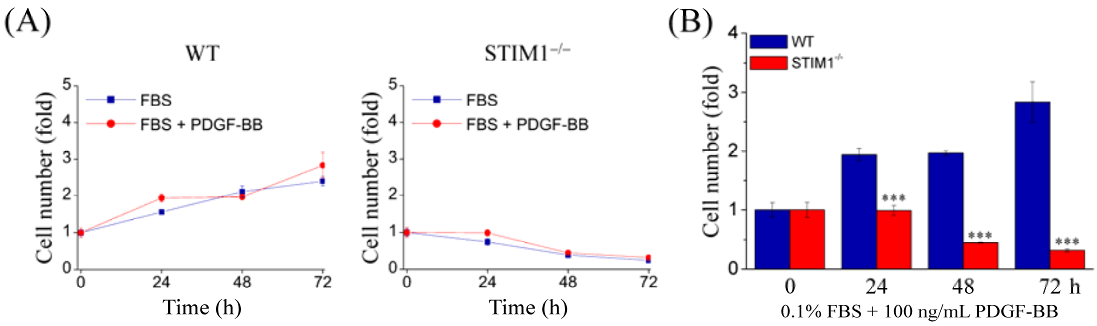

Figure S5. PDGF-BB does not affect MEF cell proliferation. (A) MEF-WT and MEF-STIM1$^{-/-}$ cells were starved in a serum-free medium for 12 h and then cultured in to DMEM with 0.1% FBS and with or without PDGF-BB (100 ng/mL) for 24, 48, and 72 h. Following fixation with 4% paraformaldehyde, cells were stained with Hoechst 33342. Fluorescence images were captured using an inverted fluorescence microscope. Cell counting was analyzed using ImageJ. Each point represents mean ± SEM from three independent experiments. (B) Comparison of the effects of PDGF-BB on cell proliferation between MEF-WT and MEF-STIM1$^{-/-}$ cells in a medium containing 0.1% FBS. ***: $P < 0.001$ by Student's *t*-test.

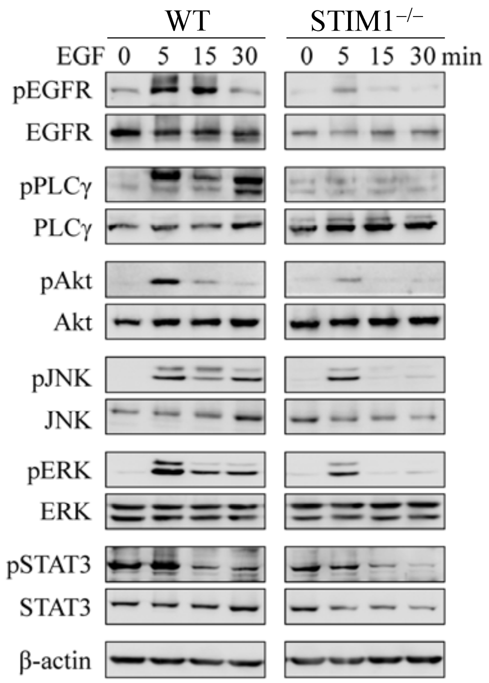

Figure S6. STIM1 knockout decreases phosphorylation of PLCγ, Akt, JNK, and ERK, but not STAT3, under EGF stimulation. MEF-WT and MEF-STIM1$^{-/-}$ cells were starved in a serum-free medium for 12 h and then stimulated with 100 ng/mL EGF for 5, 15, and 30 min. Immunoblotting analysis using antibodies against phospho-EGFR (pEGFR), EGFR, phospho-PLCγ (pPLCγ), PLCγ, phospho-Akt (pAkt), Akt, phospho-JNK (pJNK), JNK, phospho-ERK (pERK), ERK, phospho-STAT3 (pSTAT3), and STAT3. β-actin served as the internal control.
